# Transcriptional activation and coactivator binding by yeast Ino2 and human proto-oncoprotein c-Myc

**DOI:** 10.1007/s00294-025-01309-w

**Published:** 2025-01-16

**Authors:** Eva-Carina Wendegatz, Julia Lettow, Wiktoria Wierzbicka, Hans-Joachim Schüller

**Affiliations:** Center for Functional Genomics of Microbes, Institut Für Genetik Und Funktionelle Genomforschung, Universität Greifswald, Felix-Hausdorff-Straße 8, 17487 Greifswald, Germany

**Keywords:** *Saccharomyces cerevisiae*, Ino2, c-Myc, Gene activation, Transcriptional rewiring, Coactivator interaction

## Abstract

Basic helix-loop-helix domains in yeast regulatory proteins Ino2 and Ino4 mediate formation of a heterodimer which binds to and activates expression of phospholipid biosynthetic genes. The human proto-oncoprotein c-Myc (Myc) and its binding partner Max activate genes important for cellular proliferation and contain functional domains structure and position of which strongly resembles Ino2 and Ino4. Since Ino2—Myc and Ino4—Max may be considered as orthologs we performed functional comparisons in yeast. We demonstrate that Myc and Max could be stably synthesized in *S. cerevisiae* and together significantly activated a target gene of Ino2/Ino4 but nevertheless were unable to functionally complement an *ino2 ino4* double mutant. We also map two efficient transcriptional activation domains in the N-terminus of Myc (TAD1: aa 1–41 and TAD2: aa 91–140), corresponding to TAD positions in Ino2. We finally show that coactivators such as TFIID subunits Taf1, Taf4, Taf6, Taf10 and Taf12 as well as ATPase subunits of chromatin remodelling complexes Swi2, Sth1 and Ino80 previously shown to interact with TADs of Ino2 were also able to bind TADs of Myc, supporting the view that heterodimers Ino2/Ino4 and Myc/Max are evolutionary related but have undergone transcriptional rewiring of target genes.

**Supplementary Information:**

The online version contains supplementary material available at 10.1007/s00294-025-01309-w.

## Introduction

Certain families of transcription factors contain DNA-binding domains (such as zinc finger structures, basic leucine zipper and basic helix-loop-helix motifs) which are highly conserved among eukaryotes. Transcriptional activators with a basic helix-loop-helix (bHLH) structure bind DNA as homo- or heterodimers, using a four-helix-bundle formed by the HLH motifs of each monomer (two α-helices with hydrophobic residues at conserved positions, separated by an unstructured loop of variable length). Basic α-helical sequences of ~ 15 amino acids in both monomers are required for recognition of specific DNA sequences (mostly with a CANNTG core; E-box) via the major groove of the B-form DNA (mammalian Max: Ferré-D'Amaré et al. 1993; yeast Pho4: Shimizu et al. 1997). Several bHLH-proteins have been identified in the yeast *Saccharomyces cerevisiae*, responsible for fundamental functions such as activation of phospholipid biosynthesis (Ino2, Ino4), acquisition of phosphate (Pho4) and centromere segregation (Cbf1; summarized by Robinson and Lopes 2000). When precursor molecules inositol and choline are limiting, structural genes of yeast phospholipid biosynthesis are positively regulated by the heterodimeric Ino2/Ino4 bHLH complex (Schwank et al. 1995) which binds to the UAS element ICRE (inositol/choline responsive element) identified upstream of *FAS1*, *FAS2*, *INO1* and functionally related genes (Schüller et al. 1992, 1995). While both Ino2 and Ino4 are required for ICRE-binding, transcriptional activation of target genes is mediated exclusively by Ino2, containing two separate N-terminal transcriptional activation domains (TAD1, TAD2; Schwank et al. 1995; Dietz et al. 2003) which are able to interact with factors of chromatin remodeling (subunits of complexes SWI/SNF, RSC and INO80; Wendegatz et al. 2024) and basal transcription factors (subunits of TFIID and TFIIA; Hintze et al. 2017; Engelhardt et al. 2023). Nuclear import of the complex Ino2/Ino4 depends on Ino4, harboring a basic nuclear localization sequence (NLS; Kumme et al. 2008). Although genes orthologous to *INO2* and *INO4* have been identified in other yeast species, molecular functions must not be necessarily conserved. Heterologous co-expression of genes *CaINO2* and *CaINO4* from the pathogenic yeast *Candida albicans* could functionally complement the inositol deficiency of a *S. cerevisiae ino2 ino4* double mutant and CaIno2 + CaIno4 together were able to bind ICRE sequences in vitro. However, further studies provided clear evidence that *CaINO2* and *CaINO4* are not required for phospholipid biosynthesis in *C. albicans* and have instead acquired a different function (Hoppen et al. 2007). A similar “transcriptional rewiring” of regulatory networks (acquisition of a modified function for an existing transcription factor) has been described for orthologs of *INO2* and *INO4* in the oleaginous yeast *Yarrowia lipolytica* capable to utilize alkanes by induction of cytochrome P450 genes such as *ALK1* (Endoh-Yamagami et al. 2007). *ALK* genes and other genes involved in degradation of alkanes contain ARE sequence elements in their promoters (alkane-responsive element) which are bound by a heterodimer containing Yas1 (similar to Ino4) and Yas2 (Ino2). Rewiring is also evident for the genetic control of sterol biosynthesis: While most eukaryotes (including humans) use members of the bHLH-family SREBP (sterol response element binding protein) for activation of the respective genes, ergosterol biosynthetic genes in *S. cerevisiae* are regulated by the Gal4-related zinc cluster protein Upc2 (Maguire et al. 2014). Such examples demonstrate that transcriptional rewiring with a switch of binding sites and interacting regulatory proteins routinely occurs in the course of evolutionary adaptation in fungi (Rokas and Hittinger 2007). Common mechanisms also operate when developmental changes of morphological features in the evolution of invertebrates become effective (Hinman et al. 2003).

**Fig. 1:**
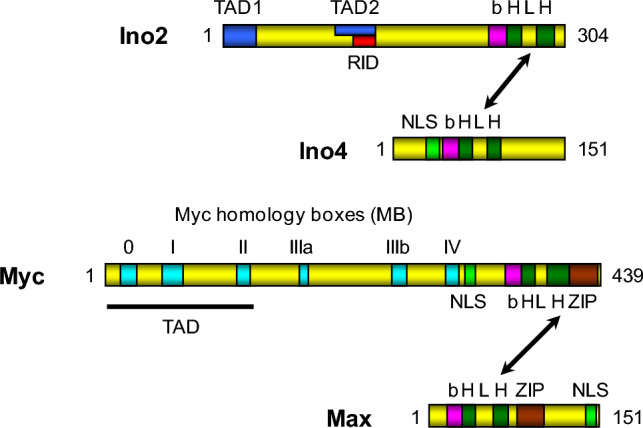
Organization of functional domains of heterodimeric transcription factors Ino2/Ino4 from *S. cerevisiae* and human Myc/Max. Position of Myc homology boxes 0-IV (MB) was taken from Cowling and Cole (2006) and Kalkat et al. (2018). *bHLH* basic helix-loop-helix (dimerization and DNA-binding), *NLS* nuclear localization sequence, *RID* repressor interaction domain, *TAD* transcriptional activation domain, *ZIP* leucine zipper

The proto-oncoprotein c-Myc (in short: Myc) is a DNA-binding transcription factor stimulating cell division in multicellular organisms and may trigger tumorigenesis upon deregulation of its biosynthesis, e. g. by genomic amplification, translocation or retroviral expression (Meyer and Penn 2008). Myc as well as its interaction partner Max contains a bHLH structural motif followed immediately by a C-terminal leucine zipper (bHLH-ZIP; reviewed by Eilers and Eisenman 2008; Lüscher and Vervoorts 2012). Together, Myc/Max may activate a large number of target genes. E-box sequences (CACRTG; R = A or G) bound by Myc/Max were found upstream of genes which stimulate cellular proliferation (cyclin-dependent kinase 4, cyclins A2, D2, E1) and metabolic functions (enolase, fatty acid synthase; Zeller et al. 2003). Conversely, Myc may also act as a transcriptional repressor (Herkert and Eilers 2010). Comparing heterodimer complexes Ino2/Ino4 and Myc/Max, a clear conservation of their molecular anatomy is apparent (Fig. 1). For dimerization and DNA-binding, Ino2 and Myc contain bHLH or bHLH-ZIP domains in their C-terminal regions while transcriptional activation is mediated by sequences at the N-terminus (TAD1 and TAD2 of Ino2 and Myc homology boxes MB0, I and II). Myc homology boxes were initially defined by bioinformatic alignment of Myc sequences (C-, N- and L paralogs) from various metazoan organisms (Cowling and Cole 2006). Using various segments of Myc fused with Gal4, Kato et al. (1990) described three transcriptional activation domains, containing MB0, I and II. In contrast to Myc, Max is able to bind CACRTG motifs as a homodimer but can also mediate gene repression by interaction with partner proteins of the Mad (Mxd) family which may subsequently recruit the pleiotropic corepressor Sin3, associated with histone deacetylases (Schreiber-Agus 1998).

The existence of similar structural motifs in Ino2 and Myc as well as in Ino4 and Max at conserved positions prompted us to perform functional comparisons, giving clues whether Ino2—Myc and Ino4—Max can be considered as orthologs which underwent transcriptional rewiring (comparison of structural predictions for Ino2 and Myc is shown as Supplementary Figure S1). We could show that Myc/Max clearly activated an ICRE-containing target gene of Ino2/Ino4 but were nevertheless unable to functionally complement an *ino2 ino4* double mutant of *S. cerevisiae*. Coactivators which had been previously shown to interact with TADs of Ino2 were also able to contact TADs mapped within Myc.

## Materials and methods

### Yeast and bacterial strains, media, growth conditions and enzyme assays

Genotypes of *S. cerevisiae* strains used in this work are shown in Table 1. Transformants of strain C13-ABY.S86 devoid of vacuolar proteinases yscA (Pep4, Pra1), yscB (Prb1), yscC (Prc1) and yscS (Cps1) were used for preparation of yeast protein extracts for in vitro interaction assays. Transcriptional activation by Gal4_DBD_-Myc fusions was assayed in transformants of strain PJ69-4A (James et al. 1996), using reporter genes *GAL7-lacZ* (activity of β-galactosidase) and *GAL2-ADE2* (growth on synthetic medium without adenine), respectively. To affinity purify GST-TAD fusions, *E. coli* strain BL21-CodonPlus(DE3)-RP (Stratagene/Agilent) containing additional tRNA genes was used. Synthetic yeast media for assaying growth of strains in the absence of phospholipid precursors inositol and choline have been described (Schwank et al. 1995). For β-galactosidase assays, strains were routinely transformed three times with plasmids and extracts from 4 transformants were investigated in each case (12 independent strain cultivations and enzyme assays).

**Table 1:** Strains of *Saccharomyces cerevisiae* used in this work

Strain	Genotype
C13-ABY.S86	*MAT*α* ura3 leu2 pra1 prb1 prc1 cps1*
JS91.15–23	*MAT* α* ura3 leu2 trp1 his3*
PJ69-4A	*MATa trp1 leu2 his3 gal4*∆ *gal80*∆ *GAL2-ADE2 LYS2::GAL1-HIS3 met2::GAL7-lacZ*
SS93.16–12	*MATa leu2 his3 trp1 ino2*∆*::LEU2 ino4*∆*::LEU2 ura3::ICRE-CYC1-lacZ::URA3*

### Plasmid constructions

A compilation of complete genotypes of expression plasmids used in this work is shown in Table S1 (Supplementary material). Human *MAX* and *MYC* were amplified by PCR with the proofreading-competent *Pwo* DNA polymerase, using specific oligonucleotides (Supplementary Table S2) and template plasmids pME562 (*MAX*) or pME920 (*MYC*) containing complete coding regions (Prof. M. Eilers, University Würzburg). PCR fragments obtained were inserted into yeast multi-copy expression plasmids and activated by the *MET25* promoter which can be derepressed in the absence of methionine (Mumberg et al. 1994). For mapping transcriptional activation domains in yeast, length variants of *MYC* were fused with the DNA-binding domain of Gal4, using plasmid pGBD-C1 (James et al. 1996). Expression plasmids with *MYC* truncations were verified by DNA sequencing (LGC Genomics, Berlin, Germany). For in vitro-interaction experiments, *E. coli* expression plasmids encoding IPTG-inducible fusions of glutathione-S-transferase (GST) with transcriptional activation domains of Ino2 (TAD1), Myc (TAD1) and Myc (TAD2) were used (Hintze et al. 2017; this work).

### In vitro interaction assays

For in vitro interaction assays, coactivators were synthesized in *S. cerevisiae* as full-length proteins or truncated variants, following the procedure described by Hintze et al. (2017). GST-TAD fusions in IPTG-treated *E. coli* transformants were released by sonication and immobilized on glutathione (GSH) sepharose beads. To ensure the use of similar amounts of GST fusions as “bait” proteins, GST enzyme assays were performed. Buffer SA-125 (20 mM HEPES, pH7.6; 1 mM dithiothreitol (DTT), 1 mM EDTA, 125 mM K-acetate, 20% glycerol, 1% NP-40) was used for incubation of bound GST-TAD fusions with total protein extracts from yeast transformants containing HA fusions of Taf1, Taf4, Taf6, Taf10, Taf12, Toa1, Swi1, Swi2, Swi3, Snf5, Snf6, Sth1 or Ino80. Yeast protein extracts were prepared by mechanical agitation in the presence of zirconia beads. Following repeated washing with PBS, bound GST fusion proteins were eluted with an excess of free GSH. Proteins were separated by SDS/PAGE, transferred to a PVDF membrane and incubated with anti-HA-peroxidase conjugate (monoclonal antibody 12CA5 conjugate; Sigma-Aldrich). Antibody-bound HA-fusion proteins were visualized by treatment of the membrane with POD chemiluminescent substrates luminol and H_2_O_2_. Luminescence signals were detected with a digital imager (ChemoStar, Intas, Göttingen, Germany). Interaction experiments were routinely performed at least twice.

## Results

### Activation of Ino2/Ino4-dependent reporter genes by Myc/Max in yeast

**Fig. 2:**
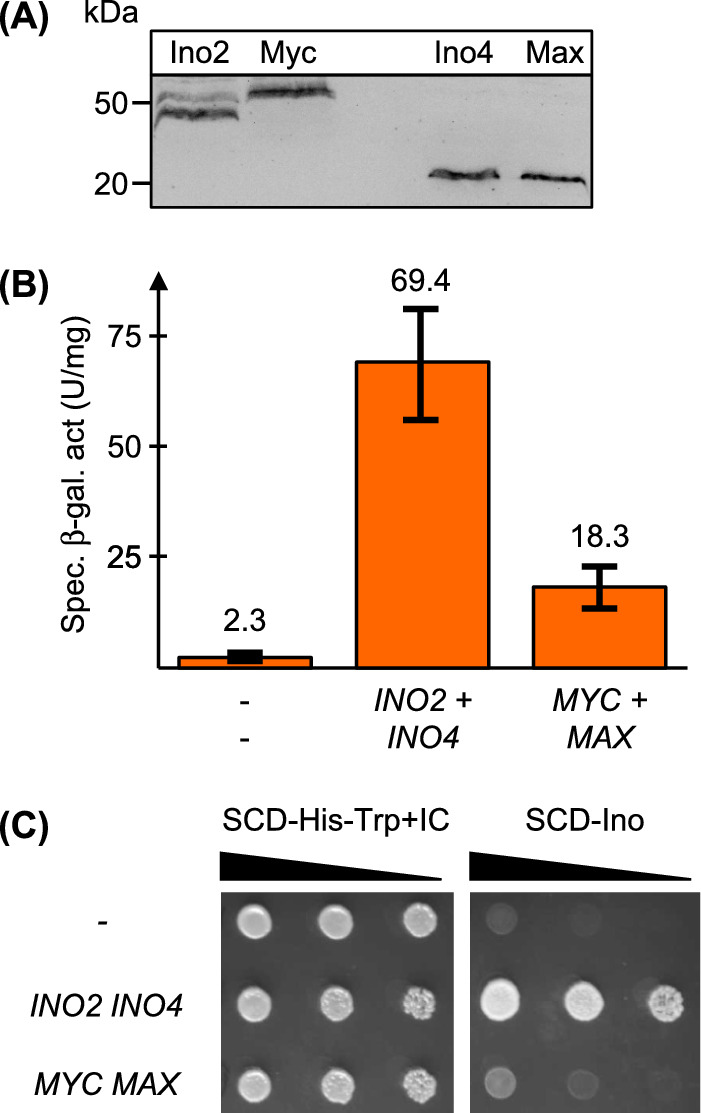
Stability and comparative functional characterization of Ino2/Ino4 and Myc/Max in yeast. **A** Myc and Max can be stably synthesized in *S. cerevisiae*. Strain C13-ABY.S86 was transformed with expression plasmids pMD2 (*MET25*_Pr_-HA_3_-*INO2*, molecular weight of fusion protein: ~ 37 kDa), pKH19 (*MET25*_Pr_-HA_3_-*MYC*, ~ 51 kDa), pMD5 (*MET25*_Pr_-HA_3_-*INO4*, ~ 20 kDa) and pKH15 (*MET25*_Pr_-HA_3_-*MAX*, ~ 20 kDa), respectively. Protein extracts from transformants were used for immunoblot analysis, using HA-specific monoclonal antibodies. To ensure comparable conditions, 50 μg of total protein was analyzed in each lane. Pr, promoter. **B** Comparison of gene expression (using integrated reporter gene ICRE-*CYC1-lacZ*) in the presence of Ino2/Ino4 or Myc/Max. Strain SS93.16–12 (relevant genotype: *his3 trp1 ura3*::ICRE-*CYC1-lacZ::URA3 ino2*∆::*LEU2 ino4*∆::*LEU2*) was doubly transformed with plasmids pJS470 (*MET25*_Pr_-*INO2*) and pJS471 (*MET25*_Pr_-*INO4*) or pKH21 (*MET25*_Pr_-*MYC*) and pKH16 (*MET25*_Pr_-*MAX*), respectively. Empty vectors p424-MET25 and p423-MET25 were used as a negative control. Transformants were grown in selective medium (SCD-Ura-His-Trp) with limiting concentrations of inositol and choline (5 µM each). Specific β-galactosidase activities (U per mg protein) were assayed in extracts prepared from double transformants which had been cultivated until mid-log phase. Strain SS93.16–12 was transformed three times with both plasmid pairs, in each case assaying extracts from 4 transformants (12 independent strain cultivations and enzyme assays). Standard deviations are indicated by error bars. **C** Assay for functional complementation of an *ino2 ino4* double mutation by *MYC*/*MAX*. Serial dilutions (fivefold) of double transformants were spotted on selective synthetic medium (− His, − Trp) supplemented with inositol + choline (left, incubation for 2 days at 30 °C) and on medium lacking inositol (SCD-Ino, right; 3 days). Growth of transformants is representative of three independent replicate experiments

It has been previously shown that co-expression of *MYC* and *MAX* in the yeast *S.* *cerevisiae* allows efficient expression of a reporter gene driven by an authentic Myc/Max binding site (CACGTG; Amati et al. 1992). Since protein pairs Ino2/Ino4 and Myc/Max show a considerable degree of similarity (Fig. 1), we wished to compare their molecular functions by studies in yeast. Coding regions of *MYC* and *MAX* were thus inserted into yeast expression plasmids, containing the *MET25* promoter for heterologous gene expression and the HA epitope for immunological detection of fusion proteins. As is demonstrated by the Western blot analysis shown in Fig. 2 A, Myc and Max could be detected in protein extracts prepared from transformants of the respective expression plasmids, indicating that Myc and Max can be stably synthesized in *S. cerevisiae*. Protein levels are similar to that of Ino2 and Ino4 which were expressed under control of the same promoter.

Although binding sites of Ino2/Ino4 (consensus sequence ICRE: WYTTCACATG; Schüller et al. 1995) and Myc/Max (canonical CACGTG motif) both contain E-box motifs CANNTG, it was unclear whether Myc/Max can bind ICRE sequences and may enable activation of yeast phospholipid biosynthetic genes. Mutational analysis of the ICRE has previously shown that Ino2/Ino4 are able to activate gene expression via TTTTCACGTG promoter sequences, although less efficiently than TTTTCACATG (Schüller et al. 1995). We thus constructed expression plasmids for *MYC* and *MAX* with distinct selection markers and transformed them into an *ino2 ino4* double deletion mutant, containing an integrated plasmid with a *CYC1-lacZ* reporter gene driven by an ICRE-dependent synthetic minimal promoter (relevant genotype: *ino2*∆ *ino4*∆ ICRE-*CYC1-lacZ*). As a positive control, corresponding expression plasmids containing *INO2* and *INO4* were used. Transformants were cultivated in selective medium with limiting concentrations of phospholipid precursors inositol and choline. The composition of this medium supports growth of *ino* mutants but allows maximal activation of the reporter gene. As is shown in Fig. 2 B, simultaneous expression of *MYC* and *MAX* in yeast substantially activates the ICRE-dependent reporter gene about eightfold above the level obtained with empty plasmids which is, however, clearly less than activation with *INO2* and *INO4* (about 30-fold activation). No activation of the reporter gene above the background level (empty expression plasmids) was observed when gene combinations *INO2*/*MAX* and *MYC*/*INO4* were co-transformed (data not shown). We also tested whether transformants containing *MYC* and *MAX* are able to grow in the absence of inositol (synthetic medium without inositol). Although the *ino2 ino4* double mutant with *MYC* + *MAX* grew slightly better than the negative control, *INO2* + *INO4* expectedly supported growth without inositol much more efficiently (Fig. 2 C). We conclude that gene activation mediated by Myc/Max compared to Ino2/Ino4 is not sufficient to stimulate expression of the biosynthetic gene *INO1*. Despite a level of ICRE-driven gene activation of 26% (*INO2*/*INO4*: 100%), *MYC*/*MAX* were unable to functionally complement the *ino2 ino4* double mutation.

### Mapping of Myc transcriptional activation domains in yeast

**Fig. 3:**
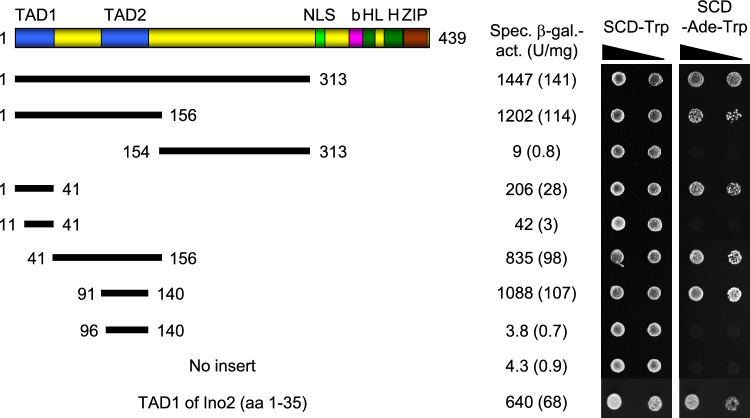
Activation of a *GAL7-lacZ* reporter gene by Gal4_DBD_-Myc fusion proteins. Plasmids pWW1 (encoding Gal4_DBD_-Myc_1-156_), pWW2 (Myc_1-313_), pWW3 (Myc_1-41_), pWW10 (Myc_11-41_), pWW4 (Myc_41-156_), pWW5 (Myc_154-313_), pWW6 (Myc_91-140_), pWW12 (Myc_96-140_) and pSH14 (Ino2_1-35_; positive control) were transformed into strain PJ69-4A (relevant genotype: *GAL2-ADE2 GAL7-lacZ*). Specific β-galactosidase activities were assayed in extracts from transformants which had been cultivated until mid-log phase. Strain PJ69-4A was transformed three times with these plasmids, in each case assaying extracts from 4 transformants (12 independent strain cultivations and enzyme assays). Standard deviations are shown in parenthesis. For qualitative growth assays, serially diluted (fivefold) transformants selected on SCD-Trp (left panel) were cultivated on SCD-Ade-Trp medium (right panel) growth on which requires restoration of a functional Gal4 variant. Transcription activation domains of Myc are depicted as defined in this work

The N-terminus of Myc (aa 1–143) comprising Myc homology boxes MB0, MBI and MBII is able to activate transcription in mammalian cells when fused to a heterologous DNA-binding domain (Kato et al. 1990). To compare transcriptional activation in mammals and in yeast, we fused length variants of Myc (aa 1–313, omitting its C-terminus with the bHLH-ZIP domain) with the DNA-binding domain of Gal4 and transformed the resulting expression plasmids into *gal4∆* strain PJ69-4A, containing Gal4-dependent reporter genes *GAL7-lacZ* and *GAL2-ADE2*. Thus, transformants display high activities of β-galactosidase and are able to grow in the absence of adenine only when a Gal4 derivative with a functional activation domain is generated. As is shown in Fig. 3, N-terminal sequences 1–313 and 1–156 of Myc mediated strong activation of the *GAL7-lacZ* reporter gene in yeast and allowed growth without adenine supplementation as a result of *GAL2-ADE2* expression. In contrast, no activation was observed with an internal length variant of Myc (aa 154–313), containing Myc homology boxes MBIIIa, MBIIIb and MBIV. Using further truncated length variants, we were able to identify aa 1–41 (designated TAD1 in Fig. 3) and aa 91–140 (TAD2) of Myc as highly efficient transcriptional activation domains in yeast. The heterologous sequence of Myc TAD2 turned out as an even more effective activation domain than the strong TAD1 of Ino2 which was used as a reference (1088 U/mg vs. 640 U/mg; Fig. 3). Further truncations of Myc TAD1 and TAD2 substantially weakened expression of the reporter gene. We thus conclude that both TAD sequences as defined in this work represent minimal domains required for transcriptional activation by Myc in yeast.

### Interaction of Myc TADs with yeast transcriptional coactivators

**Fig. 4:**
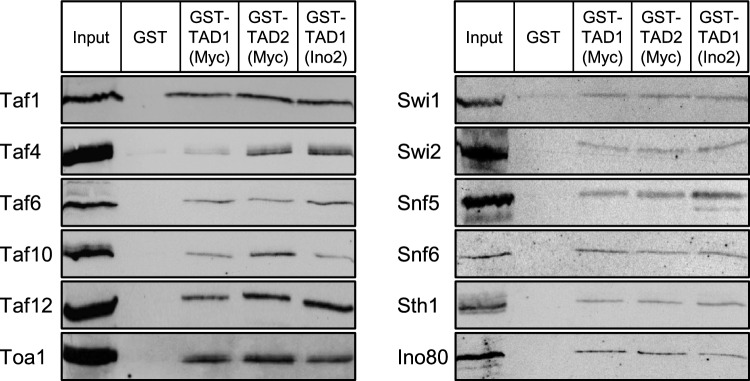
In vitro interaction assays (GST pull-down experiments) with activation domains TAD1 and TAD2 from human Myc and TAD1 from yeast Ino2. Fusion proteins GST-Myc TAD1 (aa 1–41; encoded by pWW9), GST-Myc TAD2 (aa 91–140; pWW8) and GST-Ino2 TAD1 (aa 1–35; pSH117) were synthesized in *E. coli* and bound to GSH sepharose. Empty GST vector pGEX-2TK served as a negative control. Immobilized GST fusions were incubated with yeast protein extracts containing epitope-tagged coactivators Taf1 (aa 1–250; expression plasmid pLvD1), Taf4 (pMS1), Taf6 (pEW1), Taf10 (pMS3), Taf12 (pMS4), Toa1 (pSH153), Swi1 (aa 329–657; pECW39), Swi2 (aa 1–307; pECW41), Snf5 (aa 1–334; pECW40), Snf6 (full-length, pKB1), Sth1 (aa 1–300; pECW31) and Ino80 (aa 1–670; pECW38)

TADs exert their function by forming multiple transient protein–protein interactions with coactivators which (I) increase accessibility of DNA organized in chromatin by covalent modification of histones or relocation of nucleosomes by chromatin remodelling complexes and (II) support recruitment of general transcription factors such as TFIID, TFIIA and mediator as key components of the preinitiation complex to sequences of the basal promoter (Hahn and Young 2011). We have previously shown that TADs of Ino2 interact with subunits Taf1, Taf4, Taf6, Taf10 and Taf12 of TFIID and Toa1 of TFIIA (Hintze et al. 2017; Engelhardt et al. 2023) as well as with subunits of chromatin remodelling complexes (Swi1, Swi2, Snf5 and Snf6 of SWI/SNF, Sth1 of RSC and Ino80 of INO80; Wendegatz et al. 2024). To compare coactivator recruitment by Ino2 and Myc, we performed in vitro interaction experiments (GST pull-down) with TADs fused to glutathione S-transferase (GST) and immobilized on glutathione (GSH) sepharose, assaying retention of epitope-tagged coactivators which had been synthesized in yeast as full-length proteins or truncated variants containing complete activator binding domains. As is shown in Fig. 4, all 12 coactivators which can bind to TAD1 of Ino2 were also able to interact with both TADs of Myc. Interaction of the weaker Myc TAD1 with Taf4 was reproducibly less intensive than Taf4 binding to Myc TAD2 and Ino2 TAD1. Although no sequence conservation is apparent for these TADs, the concept of a “fuzzy” interface typical for coactivators (demonstrated for TAD interaction with mediator subunit Med15; Tuttle et al. 2021) can explain the identical binding pattern observed.

## Discussion

In this work we could show that co-expression of *MYC* and *MAX* in *S. cerevisiae* allowed a significant activation of an ICRE-containing reporter gene which normally depends on Ino2/Ino4. However, expression of this gene by Myc/Max clearly remained below the level observed with authentic activators Ino2/Ino4 (26%), explaining why functional complementation of an *ino2 ino4* double mutation by synthesizing Myc/Max in yeast was not successful. In general, several reasons can explain this result: (I) Heterologous proteins may be rapidly degraded prior to execution of their function; (II) Nuclear import of heterologous proteins may be insufficient; (III) Transcription factors may fail to bind activating sequences of target genes; (IV) Transcriptional activation domains may be unable to communicate with coactivators of yeast.

Although Myc is an unstable protein in mammalian cells, our results show that both Myc and Max can be efficiently synthesized in yeast. Using multi-copy expression plasmids with the *MET25* promoter, abundance of Myc and Max was similar to homologous proteins Ino2 and Ino4. Both Myc and Max contain basic nuclear localization sequences (Kato et al. 1992; Dang and Lee 1988). Since the mechanism of importin- and Ran-dependent nuclear protein import is universally conserved in eukaryotes (Conti and Izaurralde 2001) it is plausible to assume that Myc and Max should be also nuclear in yeast. This assumption agrees with the previous finding that Myc/Max in yeast strongly activate a reporter gene with an authentic binding site (Amati et al. 1992). We thus conclude that Myc/Max and Ino2/Ino4 differ with respect to their binding site specificities.

**Fig. 5:**
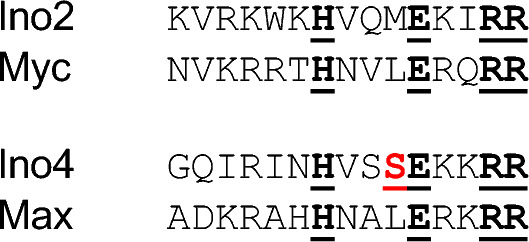
Alignment of basic regions of yeast and human bHLH proteins. Conserved amino acids making base-specific contacts to the E-box core (CACRTG) are shown underlined and in bold black letters. Serine residue S53 of Ino4 forming hydrogen bonds to T(3)T(4) of the ICRE (WYTTCACATG) is depicted underlined and in red. Structural data were taken from Ferré-D'Amaré et al. (1993), Nair and Burley (2003) and Khan et al. (2022)

Mutational analysis of individual positions within the ICRE (consensus: 5´ WYTTCACATG 3´; W = A or T; Y = C or T) clearly showed that T bases at positions 3 and 4 are absolutely required for efficient gene activation (Schüller et al. 1995). In contrast, Myc/Max discriminate against sequences with a T flanking the CACGTG core sequence as it has been shown by selection for binding sites in vitro and reporter gene assays in vivo (consensus binding sequence of Myc/Max: RACCACGTGGTY, Solomon et al. 1993). As is evident from the crystal structure analysis of ICRE-bound Ino2/Ino4, both T residues form hydrogen bonds with oxygen atoms of serine-53 (S53) within the basic region of Ino4 (Khan et al. 2022). At the corresponding position of its basic region, Max contains a leucine residue for which no DNA contacts have been described, explaining why interaction with ICRE sequences is less impactful (cf. Figure 5). Moreover, sequences outside the core of the DNA-binding domain may also influence fine-tuning of binding specificities as it has been shown for intrinsically disordered regions of yeast activators Msn2 and Yap1 (Brodsky et al. 2020).

**Fig. 6:**
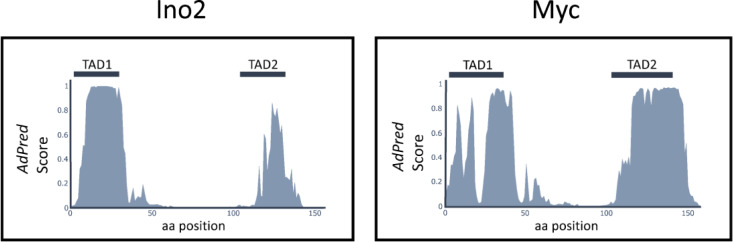
Prediction of acidic TADs for Ino2 and Myc, using the AdPred score as described by Erijman et al. 2020 (website: https://adpred.fredhutch.org). Position of Myc homology boxes within the activation region are as follows: MB0 (aa 16–33), MBI (aa 45–63), MBII (aa 128–143; Kalkat et al. 2018)

It is generally accepted that the N-terminus of Myc (aa 1–143; Kato et al. 1990) mediates transcriptional activation but different results have been obtained for the precise position of Myc transcriptional activation domains (TADs). Using chinese hamster ovary cells and Gal4-Myc hybrid proteins for their assays, Kato et al. (1990) identified three activating subdomains (aa 1–41, aa 41–103 and aa 103–143) which were considered as TADs while Flinn et al. (2002) used Myc-Pho4 fusions in yeast and mapped aa 1–41 and aa 66–127 as activation domains. Our results with Gal4-Myc fusions assayed in yeast completely agree with the position of TAD1 (aa 1–41) but modify position of TAD2 (aa 91–140). Both TADs defined in this work contain an excess of acidic amino acids and thus exhibit a negative net charge (TAD1: − 7; TAD2: − 8) together with several phenylalanine residues known to be important for efficient transcriptional activation (Erijman et al. 2020). To correlate experimental data and in silico analysis of N-terminal sequences from Ino2 and Myc, we finally used the bioinformatic tool *Adpred* developed to predict activation domains by a deep learning strategy based on a comprehensive number of verified TADs (Erijman et al. 2020). As is shown in Fig. 6, this model predicts positions of Myc TADs essentially as mapped in this work. For the core function of TAD1, Myc homology box MB0 (aa 16–33) should be essential while MBI (aa 45–63) may support activation but is not absolutely required. This interpretation agrees with mapping results presented by Kato et al. (1990) who find an activation level of only 10% for aa 41–103, compared with aa 1–41. MBI contains residues T58 and S62 which can be phosphorylated by various protein kinases in humans as well as in yeast, thus influencing Myc stability or efficiency of transcriptional activation (Escamilla-Powers and Sears 2007; Hann 2014). The very strong Myc TAD2 should definitely comprise amino acids at least until position 140, thus covering most of Myc box MBII (aa 128–143).

To stimulate initiation of transcription, TADs must transiently interact with general transcription factors (such as TFIID, TFIIA, TFIIH and mediator) finally forming preinitiation complexes (PIC) at basal promoters and also with factors of chromatin modification allowing improved access to DNA for PIC components. Importantly, the N-terminus of Myc binds to the evolutionary conserved coactivator TRRAP (Tra1 in yeast) which functions in two distinct histone acetyltransferase (HAT) complexes, STAGA (SAGA in yeast, Gcn5 as the HAT subunit) and TIP60 (NuA4 in yeast, Esa1 as HAT; McMahon et al. 1998; Frank et al. 2003; Cowling and Cole 2006; interactions are summarized by Tu et al. 2015). Importantly, TRRAP interaction was no longer possible when Myc boxes MB0 and MBII had been deleted while MBI was dispensable for Myc-TRRAP Interaction (Kalkat et al. 2018). Myc also interacts with the TRRAP-independent HAT p300/CBP (no ortholog in yeast; Vervoorts et al. 2003). Consequently, the level of target gene acetylation correlates with Myc-dependent activation. Myc also interacts with the TATA-box binding protein TBP as the core subunit of basal transcription factor TFIID and the TFIIF subunit RAP74 (McEwan et al. 1996; Wei et al. 2019). On the basis of previously identified proteins interacting with TADs of Ino2 (Hintze et al. 2017; Engelhardt et al. 2023; Wendegatz et al. 2024), we here extend the number of Myc coactivators and show that basal transcription factors Taf1, Taf4, Taf6, Taf10, Taf12 and Toa1 as well as subunits of chromatin remodeling complexes SWI/SNF (Swi1, Swi2 ATPase, Snf5 and Snf6), RSC (Sth1 ATPase) and INO80 (Ino80 ATPase) can also bind to TADs of Myc. Kalkat et al. (2018) could show that a Myc variant devoid of MBII is no longer able to interact with Taf6, Taf10 and Taf12 (which are subunits of TFIID and STAGA).

Related structural and functional domains and their conserved order among similar individual proteins do not necessarily mean that they share a common evolutionary origin. Instead, it is also possible that such proteins arose by a convergent evolution, with de novo development of functional domains under comparable selection demands. However, both Ino2/Ino4 and Myc/Max not only show an identical order of these domains and similar size but also require heterodimerization for efficient activation of target genes. Despite variation of binding site preferences and functional diversification, our results provide strong evidence that Ino2 and Myc (as well as Ino4 and Max) should be considered as orthologous activator proteins, contrary to an earlier comment (“there is no ortholog of c-Myc in yeast”; McMahon et al. 2000). This conclusion agrees with the phylogenetic classification of fungal bHLH proteins into group B also containing Myc and Max (Ledent and Vervoort 2001).

## Supplementary Information

Below is the link to the electronic supplementary material.

Supplementary file1 (PDF 79 KB)

## Data Availability

No datasets were generated or analysed during the current study.
