# Supplementary material for: Transcriptional activation and coactivator binding by yeast Ino2 and human proto-oncoprotein c-Myc
Source: Curr Genet. 2025 Jan 16;71(1):2. doi: 10.1007/s00294-025-01309-w (PMC11739200; doi:10.1007/s00294-025-01309-w)
Supplement: Supplementary file 1 — Supplementary file1 (PDF 79 KB) [file 294_2025_1309_MOESM1_ESM.pdf]

Supplementary Online Material Wendegatz et al.:

Table S1: Plasmids constructed and used in this work:

Plasmid	Genotype
p423-MET25	2µm *HIS3* (Mumberg et al. 1994)
p424-MET25	2µm *TRP1* (Mumberg et al. 1994)
p426-MET25HA	2µm *URA3 MET25*$_{Pr}$-HA$_3$ (Mumberg et al. 1994)
pECW31	2µm *URA3 MET25*$_{Pr}$-HA$_3$-*STH1*$_{1-300}$ (Wendegatz et al. 2024)
pECW38	2µm *URA3 MET25*$_{Pr}$-HA$_3$-*INO80*$_{1-670}$ (Wendegatz et al. 2024)
pECW39	2µm *URA3 MET25*$_{Pr}$-HA$_3$-*SWI1*$_{329-657}$ (Wendegatz et al. 2024)
pECW40	2µm *URA3 MET25*$_{Pr}$-HA$_3$-*SNF5*$_{1-334}$ (Wendegatz et al. 2024)
pECW41	2µm *URA3 MET25*$_{Pr}$-HA$_3$-*SWI2*$_{1-300}$ (Wendegatz et al. 2024)
pEW1	2µm *URA3 MET25*$_{Pr}$-HA$_3$-*TAF6* (full length; Hintze et al. 2017)
pGBD-C1	2µm *TRP1 ADH1*$_{Prom}$-*GAL4*$_{DBD}$ (James et al. 1996)
pGEX-2TK	tac$_{Pr/Op}$-*GST*
pJS470	2µm *TRP1 MET25*$_{Pr}$-*INO2*
pJS471	2µm *HIS3 MET25*$_{Pr}$-*INO4*
pKB1	2µm *URA3 MET25*$_{Pr}$-HA$_3$-*SNF6*$_{1-332}$ (Wendegatz et al. 2024)
pKH15	2µm *URA3 MET25*$_{Pr}$-HA$_3$-*MAX*
pKH16	2µm *HIS3 MET25*$_{Pr}$-*MAX*
pKH19	2µm *URA3 MET25*$_{Pr}$-HA$_3$-*MYC*
pKH21	2µm *TRP1 MET25*$_{Pr}$-*MYC*
pLvD1	2µm *LEU2 MET25*$_{Pr}$-HA$_3$-*TAF1*$_{1-250}$ (Hintze et al. 2017)
pMD2	2µm *URA3 MET25*$_{Pr}$-HA$_3$-*INO2*
pMD5	2µm *URA3 MET25*$_{Pr}$-HA$_3$-*INO4*
pME562	Coding region of *MAX* (Prof. Dr. M. Eilers, Würzburg)
pME920	Coding region of *MYC* (Prof. Dr. M. Eilers, Würzburg)
pMS1	2µm *URA3 MET25*$_{Pr}$-HA$_3$-*TAF4* (full length; Hintze et al. 2017)
pMS3	2µm *URA3 MET25*$_{Pr}$-HA$_3$-*TAF10* (full length; Hintze et al. 2017)
pMS4	2µm *URA3 MET25*$_{Pr}$-HA$_3$-*TAF12* (full length; Hintze et al. 2017)
pSG14	2µm *TRP1 ADH1*$_{Prom}$-*GAL4*$_{DBD}$-*INO2*$_{1-35}$ (TAD1)
pSH117	tac$_{Pr/Op}$-*GST-INO2*$_{1-35}$ (TAD1) (Hintze et al. 2017)
pSH153	2µm *URA3 MET25*$_{Pr}$-HA$_3$-*TOA1* (full length; Engelhardt et al. 2023)
pWW1	2µm *TRP1 ADH1*$_{Prom}$-*GAL4*$_{DBD}$-*MYC*$_{1-156}$
pWW2	2µm *TRP1 ADH1*$_{Prom}$-*GAL4*$_{DBD}$-*MYC*$_{1-313}$
pWW3	2µm *TRP1 ADH1*$_{Prom}$-*GAL4*$_{DBD}$-*MYC*$_{1-41}$
pWW4	2µm *TRP1 ADH1*$_{Prom}$-*GAL4*$_{DBD}$-*MYC*$_{41-156}$

pWW5	2µm *TRP1 ADH1*$_{Prom}$-*GAL4*$_{DBD}$-*MYC*$_{154-313}$
pWW6	2µm *TRP1 ADH1*$_{Prom}$-*GAL4*$_{DBD}$-*MYC*$_{91-140}$
pWW8	tac$_{Pr/Op}$-*GST-MYC*$_{91-140}$ (TAD2)
pWW9	tac$_{Pr/Op}$-*GST-MYC*$_{1-41}$ (TAD1)
pWW10	2µm *TRP1 ADH1*$_{Prom}$-*GAL4*$_{DBD}$-*MYC*$_{11-41}$
pWW12	2µm *TRP1 ADH1*$_{Prom}$-*GAL4*$_{DBD}$-*MYC*$_{96-140}$

Pr, promoter; Op, operator; all positions refer to the protein encoded by the gene given.

Table S2: Oligonucleotides used (PCR primers for amplification of *MAX* and *MYC*, construction of length variants for *MYC*):

**Name**	**Gene**	**Position**	**Sequence 5'-3'**
Max-Bam	*MAX*	-3/+20	gact**ggatcc**ATAATGAGCGATAACGATGACAT
Max-Hind	*MAX*	+483/+461	gact**aagctt**TTAGCTGGCCTCCATCCGGAGCT
cMyc-Bam	*MYC*	-3/+20	gact**ggatcc**ATAATGCCCCTCAACGTTAGCTT
cMyc-Hind	*MYC*	+1320/+1301	gact**aagctt**TTACGCACAAGAGTTCCGTA
cMyc-Bam_1	*MYC*	+1/+20	gact**ggatcc**ATGCCCCTCAACGTTAGCTT
cMyc-Bam_11F	*MYC*	+31/+50	gact**ggatcc**AACTATGACCTCGACTACGA
cMyc-Bam_41F	*MYC*	+121/+140	gact**ggatcc**CAGCCCCCGGCGCCCAGCGA
cMyc-Bam_91F	*MYC*	+271/+290	gact**ggatcc**GGGAGCTTCTCCACGGCCGA
cMyc-Bam_96F	*MYC*	+286/+305	gact**ggatcc**GCCGACCAGCTGGAGATGGT
cMyc-Bam_154F	*MYC*	+460/+479	gact**ggatcc**GCTGCGCGCAAAGACAGCGG
cMyc-Hind_41R	*MYC*	+123/+104	gact**aagctt**CTACTGCAGCTCGCTCTGCTGCT
cMyc-Sal_140R	*MYC*	+420/+401	gact**gtcgac**CTAGGCCGAGAAGCCGCTCCACA
cMyc-SalA_156	*MYC*	+468/+449	gact**gtcgac**CTAGCGCGCAGCCTGGTAGGAGG
cMyc-SalB_313	*MYC*	+939/+920	gact**gtcgac**CTAGGGAGGCGCTGCGTAGTTGT

Artificially inserted cleavage sequences for restriction enzymes are shown in **bold**; capital letters represent genuine gene-specific sequences; artificial stop codons are underlined.

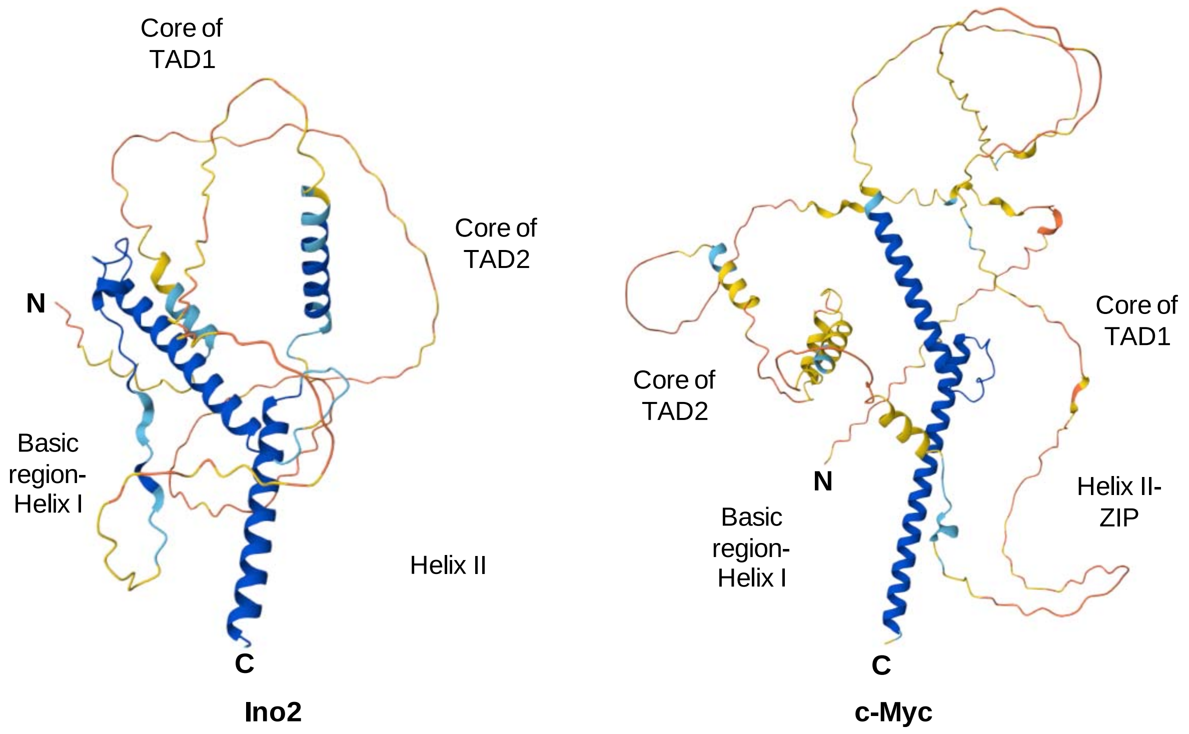

Supplementary Fig. S1: Comparison of AlphaFold-generated structural predictions for Ino2 and c-Myc. DNA-binding domains (basic-helix-loop-helix or basic-helix-loop-helix-leucine zipper) and core sequences of transcriptional activation domains are indicated by black arrows.
